# Development, feasibility testing and perceived benefits of a new app to help with adherence to antiretroviral therapy in people living with HIV in Brazil

**DOI:** 10.1186/s40814-023-01370-7

**Published:** 2023-07-26

**Authors:** Bruno Luis Schaab, Eduardo Remor

**Affiliations:** Institute of Psychology, Universidade Federal Do Rio Grande Do Sul, Rua Ramiro Barcelos, 2600, Porto Alegre, RS. 90035003 Brazil

**Keywords:** App development, HIV, Adherence, Treatment, Feasibility

## Abstract

**Background:**

Despite the availability of antiretroviral therapy (ART) in many countries, people living with HIV still experience difficulties with treatment. We propose a new smartphone mobile application to assist in adherence to ART. This study aimed to describe this new mobile application’s development (content construction and usability), feasibility testing (recruitment, retention rates [attendance], satisfaction) and primary perceived benefits.

**Methods:**

Two consecutive studies were conducted. First, people living with HIV, health care workers and experts in information technology provided feedback to improve the content and usability of the app. After changes in the app were implemented according to the feedback, a second study was performed to assess the feasibility and perceived benefits. Effects on self-reported adherence and perceived well-being were also assessed.

**Results:**

Scores of participants (*N* = 11) showed differences in adherence (effect size .43) and well-being (effect size .45) after using the app. However, the differences did not reach statistical significance. Observing scores individually, six out of 11 participants improved their overall adherence scores, and seven out of 11 participants improved their perceived well-being scores. Recruitment was 95%, and attendance at sessions was 62.5%. In general, the participants were satisfied with the intervention and viewed the app as an informative tool.

**Conclusion:**

The results are promising and allow us to recommend further studies with the app.

**Supplementary Information:**

The online version contains supplementary material available at 10.1186/s40814-023-01370-7.

## Key messages regarding feasibility

- Mobile applications are a promising solution for addressing nonadherence to HIV medication. However, a mobile application has not been tested in the Brazilian context, and it is unclear whether it is appropriate to address low-resource populations with detectable viral load.
- This study’s key findings on feasibility were as follows: qualitative feedback on the app helped improve the content. Feedback was primarily positive, indicating its applicability and user satisfaction. The recruitability worked as expected; the app elicited satisfaction and positive perceptions. The app did not reduce adherence levels or perceived well-being compared to baseline levels. The study showed promising results, indicating the app’s potential effectiveness for further controlled studies.
- The following implications will be considered in the design of the main study: recruiting a large group of participants, including both low- and medium-resource populations with detectable viral load, and implementing the adjustments to the app based on late feedback to improve user independence.

## Introduction

The launch of antiretroviral therapy (ART) is one of the main milestones in battling and controlling HIV infection and AIDS. These medications enable greater control of the viral load, promoting health maintenance and quality of life [1] and contributing to the reduction of mortality and morbidity [2, 3]. ART decreases the transmission of the virus because people with an undetectable viral load stop transmitting HIV [4]. Therefore, the correct and assiduous intake of these medicines is essential.

For different reasons, however, nonadherence to ART may be a shared experience among people living with HIV. The barriers to its access and compliance can be diverse, varying from social aspects such as poverty, hunger and food insufficiency [5] to the complexity involved in the treatment (the quantity of pills ingested, therapeutic scheme, side effects) and psychological factors [6], such as the presence of depressive symptoms and stress [7].

Self-care tools in health have been described as a suitable means to assist in adherence to the treatment of chronic diseases and to address barriers described in the literature. These tools enable patients to acquire and execute new skills and to take an active role in their treatment. In the case of HIV, tools for the development of skills in carrying out treatment have been described as relevant to results [8].

One way to prevent the problem of nonadherence to treatment in chronic diseases is digital interventions [9]. These interventions allow for personalized and available content 24 h a day in addition to having a better cost benefit than face-to-face interventions [10]. With the spread of mobile phones with internet access, the mHealth field has been consolidated, and mobile tools are more widely used to host interventions in the field of health [11]. Such technologies can serve as hosts and personalized self-care tools [12], and their acceptability [13] and effectiveness have been described [14], including for HIV and AIDS [8, 9, 15]. Apps launched in the context of adherence to ART have shown evidence of their effectiveness for different audiences, such as men who have sex with men [15], substance users [16] and youths [17].

Despite the spread of mHealth technologies, not all apps are built from a theoretical perspective or use scientific techniques to change behaviour, which can weaken the intervention’s potential. The use of theoretical frameworks is associated with larger effect sizes in digital interventions [18], which suggests the importance of this aspect in the development of these interventions. Moreover, many ART apps are built and sold without being tested for their benefits or acceptance by users [11].

## The development of the new smartphone application: “ + Adesão!”

To develop a new smartphone application (“ + Adesão!” in the original Portuguese, meaning “plus adherence”), we followed the approach described by Bradbury et al. [12], starting with intervention planning followed by intervention development and usability testing and finishing with intervention testing. We chose to focus the activities of the app in the domains of compliance, antecedents of nonadherence behaviours, doctor–patient communication, personal beliefs/expectations about treatment and treatment satisfaction. These domains have been reported to be related to adherence behaviour [6] and are included in a measure of widespread adherence to ART in Latin America [19] called the Questionnaire to Evaluate the Adherence to HIV Therapy (CEAT-VIH is the acronym in the original study) [20, 21].

We propose a digital intervention based on a cognitive-behavioural approach that focuses on each of the previously described dimensions with the aim of improving adherence to ART. Initially, behaviour change techniques used in the health area were identified. Then, activities based on these techniques were designed, which we call thematic modules. The research team, including psychologists and a research assistant in the area of computer engineering, devised the content, format and presentation of version one of the mobile application.

The computer engineering research assistant was responsible for implementing the content and exercises into the tool. The first version was developed in a hybrid programming language using the IOS system. This programming language was chosen due to the previous experience of this research assistant. The hybrid language allowed the app to be easily adapted to another platform, such as Android. The tool was designed to provide discretion, anonymity and confidentiality.

To use the app, the user must initially register and then log in. First, the user responds to an assessment protocol (i.e. the CEAT-HIV) and receives visual and written feedback on his or her adherence behaviour to treatment. The user is also assigned to activities according to his or her adherence levels. The app sorts and presents the modules to the user starting from the domain in which the user scores lowest, i.e. the domain with which the user experiences the most difficulty. The app prioritizes areas where the user has an urgent need to improve. After completing all modules, the user is assessed again and receives new feedback indicating whether he or she has improved in each of these dimensions. A description of the modules in the “ + Adesão!” app and its theoretical structure according to the behaviour change technique (BCT) taxonomy [22] are presented in Table 1. Further information is available at the TIDieR (Template for Intervention Description and Replication) Checklist; see Appendix 1.

**Table 1:** “ + Adesão!” modules and theoretical structure according to the BCT taxonomy

**Module**	**Objective**	**BCT taxonomy**^**a**^
Becoming aware of the level of adherence to treatment	Make users aware of their degree of adherence to treatment by the CEAT-HIV score	2.2. Feedback on behaviour
Information about and importance of adherence to treatment	Make users expand their knowledge of HIV and treatment	4.2. Information about antecedents
Treatment beliefs and expectations (negative thoughts and behaviour)	Assist in restructuring dysfunctional beliefs about treatment	13.2. Framing/reframing
Treatment beliefs and expectations (developing positive treatment expectations)	Assist the user in building more positive beliefs about treatment	13.2. Framing/reframing
		4.3. Reattribution
Communication with health professionals	Promote the improvement of users’ communication with doctors and health professionals regarding their treatment	4.1. Instructions on how to perform the behaviour
		2.2. Feedback on behaviour
Dealing with unforeseen events in treatment	Reflect, instruct and develop skills for handling unforeseen events in the course of treatment	5.1. Information about health consequences
		9.2. Pros and cons
		9.3. Comparative imagining of future outcomes
		4.1. Instructions on how to perform the behaviour
Feedback on the level of adherence to treatment after intervention completion	Make the user aware of changes in the degree of adherence to treatment by comparison of CEAT-HIV scores before and after the use of the app	2.7. Feedback on outcome(s) of behaviour

*Note*: ^a^http://www.bct-taxonomy.com/

## The present study

According to the literature, for consistent development of the app, it is relevant to obtain data to debug it to improve its usability, presentation, content and format. In this sense, end users, health professionals and information technology experts can provide feedback to assist in the construction of the tool. With those steps, there may be greater acceptance of the technology [12]. The main aim of the first study was to develop the content of the app, gather suggestions for improving the content, presentation and usability of the app, and gather general feedback about the app. In preparation and as antecedents for randomized clinical trials, pilot and feasibility studies were suggested [12, 23]. In this case, assessing the effectiveness of the intervention was not the most important issue, but some components of this assessment will contribute to adjusting the preparation for a more robust study in the future, such as recruitability, attendance and acceptability. Thus, the objectives of the second study were to assess the results for the “ + Adesão!” app concerning recruitability, attendance, general perceptions and satisfaction. Additionally, we wanted to evaluate the potential benefits of the app to promote adherence to antiretroviral therapy and to ensure that the use of the app did not damage well-being.

## Study 1

### Usability, content assessment and feedback for the development of the “ + Adesão!” app

#### Methods

##### Participants

The study used a person-based approach [24] in which end users of the app (people living with HIV), experts in the area of information technology (informatics) and health care workers were invited to participate to focus on understanding and accommodating the perspectives of potential users of the app. The study had 24 participants with roles in the development of the “ + Adesão!” app. The participants were arranged in distinct groups: 13 experts working in HIV health services (i.e. one psychologist, one pharmacist, four physicians, five nurses and two auxiliary nurses), three information technology experts with previous experience in web application development (i.e. one web developer, one professor/researcher and one computer scientist) and eight people living with HIV (age range 22 to 65 years old). Participants were recruited through the public health care centre and the university. In all cases, the selected sample was nonprobabilistic [25] because participation was based on the availability and interest of the participants.

##### Instruments and measure

Due to the different aims of the study, different measures were used according to the role of each part of the sample in the review and development of the app.

##### Health care professionals and informatics experts

- *Sociodemographic questionnaire*—The sociodemographic questionnaire was developed ad hoc for this study and included information on the participants’ degree (academic background), time of professional experience with HIV, experience with development of digital technologies, and weekly work hours spent caring for people living with HIV.
- *Perceptions about the app questionnaire*—This questionnaire was developed ad hoc for the study to collect information on six items addressing participants’ perceptions of the app, with a response format of a visual analogue scale from 0 to 100 mm. These items related to dimensions such as relevance, usefulness, future possibility of using the app by people living with HIV, ease of use and adequacy of the active components of the intervention. The authors considered scores above 70 as a criterion for indicating relevance, usefulness and usability.
- *Interview script for qualitative assessment*—This interview script was developed ad hoc to guide interviews with experts regarding their impressions of the app and to collect suggestions and general comments.

##### People living with HIV

- *Sociodemographic questionnaire*—This questionnaire was developed ad hoc for this study and included information on gender, education level, race/ethnicity, income, marital status and age.
- *System Usability Scale (SUS)*—This measure developed by Brooke [26] is a simple ten-item scale that provides a global view of subjective assessments of usability. It also provides an objective score regarding the usability of some digital applications, such as smartphone applications, and is frequently used in the field of software engineering. The questions are answered on a Likert scale from 1 (strongly disagree) to 5 (strongly agree). To calculate the SUS score, the score contributions from each item are summed. Each item’s score contribution ranges from 0 to 4. For items 1, 3, 5, 7 and 9, the score contribution is the scale position minus 1. For items 2, 4, 6, 8 and 10, the contribution is 5 minus the scale position. The sum of the scores is multiplied by 2.5 to obtain the overall value of the SUS, which varies from 0 to 100. Scores above the cut-off point of 68 represent good usability.
- *Utility, Satisfaction, Ease of Use* *and Ease of Learning Questionnaire*—The questionnaire is an ad hoc tool developed for this study to complement the measure of usability. The brief ad hoc questionnaire includes four questions that assess the utility, satisfaction, ease of use, and ease of learning how to use the application (see questions in Table 5). Each of these parameters is scored from 0 to 10 on a Likert scale. The authors considered scores above 7 (on a scale from 0 to 10) as criteria for achieving utility (usefulness), satisfaction, ease of use (usability), and ease of learning. In addition, this measure includes two additional open-ended questions about the strengths and weaknesses of the app.

##### Data collection and procedures

Health care workers and informatics

After approval was obtained from the Research Ethics Committee (CAAE: 14421219.5.0000.5334), a health care centre was contacted, authorizations were obtained, and a formal meeting with health care workers to evaluate the app at their work facility was arranged. During the meeting, the research team presented the “ + Adesão!” app to them and requested their opinions and evaluation. All participants gave their consent to participate and signed an informed consent form. The meeting duration was approximately 1 h 45 min.

On this occasion, the data collection procedure was performed. Through slides, one of the researchers presented the smartphone application, emphasizing its format and active components. Next, the participants were able to use and examine the “ + Adesão!” app. Open-ended questions were asked based on the established script. In addition, participants completed the questionnaire on perceptions of the app.

On a different day, the second stage of the research was conducted with informatics. Five potential study participants were contacted by email and invited to participate. Informatics experts were interviewed individually due to their work schedule and to allow the assessment to be personalized and in depth. All gave their consent and signed the informed consent form.

These experts used the app as they answered questions about it, provided their feedback, and answered the perceptions questionnaire. One expert did not provide useful feedback (only comments unrelated to the app) and did not complete the perceptions questionnaire. The feedback data reached saturation in the third interview, fulfilling the criterion of qualitative data closure. This phase ended with three participants.

Participants living with HIV

After the HIV/AIDS health care centre was contacted and the authorizations were obtained, a trained researcher was introduced by the coordinator of the centre to patients who were in the waiting room for a routine medical consultation. The researcher approached each of these patients individually and talked about the research in more detail, asked for their voluntary participation and requested that they sign a consent form. Participants who agreed to participate used the “ + Adesão!” app for 30 min on average in a private room of the facility.

At the end of their use of the app, the participants answered the sociodemographic questionnaire, the SUS and the Utility, Satisfaction, Ease of Use and Ease of Learning Questionnaire. The researcher interviews asked patients to report their experience using the app. These interviews aimed to identify the app’s strengths and weaknesses.

##### Data analysis

All sociodemographic data and the quantitative measures used were tabulated and analysed using the Statistical Package for Social Sciences, version 18.0 (SPSS® Inc, Chicago, IL). Quantitative measurements were represented by means, standard deviations and ranges. Sociodemographic variables were represented in terms of frequencies and percentages. Descriptive data resulting from the interview for qualitative assessment and open-ended questions about the strengths and weaknesses of the app were tabulated separately to obtain helpful information to implement changes in the app. In this case, the procedure used was a direct qualitative synthesis and a complete description of the themes.

#### Results

Table 2 addresses the qualitative feedback (perceptions) for the app. Overall, the app received positive comments from both experts and people living with HIV. The app was considered a tool that allows anonymity, can be a motivator in treatment and helps individuals understand the importance of adhering to ART. People living with HIV stressed that the app was easy to use and featured some interesting content. However, some criticisms noted barriers such as a large amount of text to read, lengthy content and the need for self-motivation and discipline.

**Table 2:** Qualitative feedback (general perceptions about the app)

Qualitative feedback and comments provided
**Health care workers and informatics (*n* = 16)**
The app allows anonymity
Adherence assessment is done in a playful way, which is a positive point
The app can help increase understanding of the importance of adherence
May be a motivator in treatment
The app provides space for those who do not like to share their feelings with health professionals
It is a useful and necessary tool
The need to have an email address is a barrier to app use
Self-motivation and discipline can be a problem for some people
The content of the app is too long and requires many writing tasks
**People living with HIV (*n* = 8)**
The app is relaxing to use
Presents interesting texts about mood and depression
Helpful app for those who have not started treatment
The app is easy to use
Some questions do not make sense
There is too much text

Table 3 provides a better understanding of the expert evaluation of the “ + Adesão!” app. Considering that the criteria established a priori by the authors to determine the sufficiency of some aspects was 70 points out of the maximum possible score (100) for each item, three items met these criteria (i.e. a, b and c), two items were near 70 points (i.e. e and f), and one item with a mean response above the middle of the scale (i.e. d) did not achieve the expected score. Globally, according to all reactions, none of the results indicated a negative assessment of the app in terms of usefulness, relevance, ease of use by the targeted audience and adequacy to assist in adherence. These results allow us to claim that the app was seen as helpful to assist in HIV treatment and was considered a relevant and valuable tool. Although some aspects of perceptions about the app (e.g. being easy to use by people living with HIV) were not evaluated as expected by a few health care professionals interviewed at this point, this may be related to the presuppositions of some professionals in terms of patients’ capacity and autonomy for self-care. Further results, such as those described in Table 5, show that when patients were asked about the ease of using the app, they answered that it was very easy to use.

**Table 3:** Perceptions about the app: quantitative feedback, *N* = 16

**Perceptions about the app questionnaire**	Mean^a^	(Min–Max)	Standard deviation
Question (response format)			
(a) The app can be useful to assist in HIV treatment (0 not useful–100 very useful), *n* = 15	82.63	33–100	20.59
(b) In your opinion, the app is relevant and seems useful (0 not relevant–100 very relevant)	88.79	70–100	12.43
(c) The app is relevant compared with other digital resources (apps and digital interventions) aimed at those living with HIV (0 not relevant–100 very relevant), *n* = 13	76.99	43–100	20.55
(d) In your opinion, the app will be easy to use by people living with HIV (0 not easy–100 very easy), *n* = 15	54.96	17–80	14.66
(e) In general, the content of the intervention (active components) is adequate to assist in treatment adherence (0 not adequate–100 highly adequate), *n* = 13	68.11	47–100	16.53
(f) In your opinion, how likely is the app to be used by the target audience when available? (0 not used–100 highly used)	66.7	31–100	17.12

^a^A score above 70 is expected as a criterion for achieving relevance, usefulness and usability

Table 4 indicates all participants’ suggestions for app improvement. The research team made decisions about which feedback suggestions were relevant to apply at the present moment considering the objectives of the app and the target audience. Informatics made the most relevant contributions to improving the usability of the app, making navigation easier and making the “ + Adesão!” app more intuitive by inserting control bars, more back buttons and careful colour semantics. The group of people living with HIV made very few suggestions for content improvement and usability. The majority of health care workers’; suggestions focused on broader themes unrelated to adherence to medication or suggestions for features already covered by other apps.

**Table 4:** Qualitative feedback (suggestions to improve the app)

Qualitative feedback and suggestions provided	Implementation before pilot test (App 1.0)
**Health care workers**	
Provide relevant links, government materials and links to scientific pages that provide medical information on HIV and AIDS	No
Include information about the interaction of HIV medicines and drug use	No
Decrease the number of topics that require user writing	Partially
Enter medication reminders to allow the app to guide intake of doses	No
Reduce the amount of information in the app (questions and answers)	No
Use more back buttons (return option)	Yes
**Informatics**	
Use blue or lighter colours in the text	Yes
At the assessment module, indicate how many questions there are and how many are left to the end	Partially
Put up a screen that explains how the app works	No
Improve the layout of the text and content	Yes
At the adherence feedback screen, adjust the proportion between dimension and feedback spaces	Yes
In the date function for writing the journal, use only valid days	No
When the app suggests a set of behaviours for the user to select, adjust text size	Yes
Increase the size of the text box and font when prompted to type something	Yes
Review the final part of the module as four questions are asked but only two answer options are offered (yes or no)	No
Include a stop button for the audio player	Yes
When the module presents strategies for dealing with unforeseen issues, highlight in bold or italics the most important points	No
Centre the icon of the “plus” button in the advantages and disadvantages section	Yes
Dub the video that talks about HIV and AIDS	No
The thoughts the user writes should always be presented again when a question is asked	No
Allow the user to speak rather than write	Yes
Put pop-up windows in the menu with topic information	Partially
Demarcate information that is required in registration	Yes
Put control bar on audio player	Yes
Put visual feedback on modules already made and to be made	Yes
Put progress bar on each module	Yes
Enter the minimum number of characters in the password	Yes
Indicate that the audio and text displayed are the same	No
Notify what is enabled and disabled in the main menu using colour semantics	Yes
Conduct performance tests in the future using the Android platform	No
Provide content or feedback that can be motivational	No
Prepare the app for adverse conditions (internet crash and app exit)	No
**People living with HIV**	
Include information about the daily life of people living with HIV (for example, going to get a manicure)	No
Decrease the amount of text and increase the number of images	Partially
Share external social networking links about living with HIV	No
Place the option “does not apply” on the doctor–patient relationship issue list	Yes

Overall, 15 out of 36 feedback suggestions were fully implemented (41.7%), and five were partially implemented (13.9%). Regarding the groups of participants, health care workers made six suggestions, but only one of these was implemented (16.7%) and one was partially implemented (16.7%). Informatics made 26 suggestions, of which 14 were fully implemented (53.8%) and two were partially implemented (7.7%). Finally, people living with HIV made four suggestions, and two were partially implemented (50%).

Table 5 compiles the results of all usability measures (questionnaires) applied to the participants living with HIV. As shown in the table, the means of items related to facilitators of usability on the SUS questionnaire were above 4.0, which indicates good usability. Additionally, items related to barriers to usability were below 2.3, which also shows good usability. The calculated overall value of the SUS questionnaire for the eight respondents was 89.07, above the minimum expected of 68, indicating good usability.

**Table 5:** Usability measures: system usability scale and utility, satisfaction, ease of use and ease of learning questionnaire

Item/Instrument	Mean	(Min–Max)	Standard deviation
**System Usability Scale (*n* = 8)**			
I think that I would like to use this system frequently	4.00	2–5	1.19
I found the system unnecessarily complex	1.88	1–4	1.24
I thought the system was easy to use	4.75	3–5	0.70
I think that I would need the support of a technical person to be able to use this system	1.13	1–2	0.35
I found the various functions in this system were well integrated	4.88	4–5	0.35
I thought there was too much inconsistency in this system	1.00	1–1	0
I would imagine that most people would learn to use this system very quickly	4.63	3–5	0.74
I found the system very cumbersome to use	1.25	1–3	0.70
I felt very confident using the system	4.88	4–5	0.35
I needed to learn a lot of things before I could get going with this system	2.25	1–5	1.83
**Utility, Satisfaction, Ease of Use and Ease of Learning Questionnaire (*n* = 8)**			
On a scale from 0 to 10, in your opinion, indicate the general utility of the “ + Adesão!” app (0 useless–10 very useful)	9.63	9–10	0.51
On a scale from 0 to 10, in your opinion, indicate your general satisfaction with the “ + Adesão!” app (0 dissatisfied–10 totally satisfied)	9.50	8–10	0.75
On a scale from 0 to 10, in your opinion, how easy was it generally to use the “ + Adesão!” app? (0 difficult to use–10 very easy to use)	9.88	9–10	0.35
On a scale from 0 to 10, in your opinion, how easy was it generally to understand and learn to use the “ + Adesão!” app? (0 difficult to learn to use–10 very easy to learn to use)	9.75	9–10	0.46

Likewise, the complementary usability measure (i.e. Utility, Satisfaction, Ease of Use and Ease of Learning Questionnaire) had means above 9.50. These results show the app’s potential in terms of user experience and refer to the ease of use of the app. Based on the results, we can assume that version 1.0 of the app had a good level of usability.

#### Discussion

The present study has some strengths in guiding app development via a user-centred method and incorporating all stakeholders in the process of developing the mHealth tool. The usability of the content of the app was evaluated by different participants, and the synthesis of all results aimed to guide changes in the app and improve it. Furthermore, the developers of the “ + Adesão!” app implemented suggestions made through qualitative feedback according to their applicability and practicability. The quantitative scores of the instruments also provided information on the usability and content of the app.

The following discussion is presented according to the iterative steps we performed. First, we reflect on the perceptions of the app provided by experts (informatics experts and health care workers). Additionally, we discuss the feedback to improve the app and the ways in which we implemented the suggestions. Finally, we reflect on the usability elements from the end-user perspective.

#### General perceptions of the app

It is worth emphasizing that the assessment of the app by health care workers and informatics experts indicated that the tool was useful and relevant and had the potential to serve as an adjuvant to medical treatment. We hypothesized that the app was seen in this way due to the importance of the nonadherence problem in our context [27, 28] and the lack of tools or skills to address this problem.

Another critical point is the widespread use and consolidation of smartphones in Brazil. According to the national Survey on the Use of Information and Communication Technologies in Brazilian Households [29], approximately 83% of the Brazilian population has at least one mobile phone, and 74% of these individuals access the internet through this device. Phones and tablets have served as a means of hosting digital tools focused on physical and mental health, constituting an intervention space called mHealth. Therefore, mHealth may be seen as a potential means for health promotion practices.

When compared to other existing digital apps and features, the “ + Adesão!” app was evaluated as relevant. A recent literature review highlighted the spread of apps in the context of HIV/AIDS; however, only 60% of apps were designed to improve adherence to treatment, and 44% were constructed under some behaviour theory [8]. The fact that this app addresses the intervention with variables identified in a well-known measure in our context (domains of the CEAT-VIH) may have positively impacted this assessment.

On the other hand, experts indicated that the app might not be user-friendly for all patients. Some possible reasons for this evaluation lie in the perception of excessive content to be read and written. Since people living with HIV can be stigmatized in different ways by health care workers [30–32], we hypothesized that their perceptions on this matter might be related to their view and presumption of HIV patients as having less education or skills with regard to digital technology.

Regarding the potential for using the app by end users when available, experts noted some reservations. Evidence indicates that HIV diagnosis can lead people to poverty through stigma [33], which could prevent access to mobile devices and the internet. Furthermore, experts claim that it is difficult to change health behaviours and self-care because this involves self-motivation and discipline. Apps focusing on health prevention and promotion are often downloaded and installed, but end users are likely to not follow up with them [34]. The opinion of some experts regarding the probability of non-use may be due to the intervention’s active components; when experts were not familiar with them, they could not understand their validity. In addition, with respect to active components for behaviour change, one of the possible obstacles may be the excessive reading and writing elements that constitute the intervention (the app). According to feedback from health care workers, many patients have difficulties in terms of literacy. However, there was no specific criticism of behaviour change techniques in the qualitative feedback but rather in how they were displayed.

#### Feedback to improve the app

Health care workers, informatics experts and people living with HIV made suggestions to improve the app. These contributions were related to the usability and content of the “ + Adesão!” app. As expected, most informatics feedback was related to usability, while health care workers made contributions that addressed usability and content, and people living with HIV made a few suggestions. Additionally, each group underwent a separate usability assessment, and these aspects are discussed in the next section.

The feedback was implemented after a research team meeting that determined that changes were to be made according to their applicability and relevance. The feedback was organized into three different categories: fully implemented feedback (yes), partially implemented feedback or resolution of the underlying problem in another way (partially), and non-implemented feedback (no).

Informatics specialists made relevant suggestions regarding human–computer interaction that served to improve the app’s usability. These elements are essential as digital interventions tend to be most effective when the potential for affordable use is valued and problems with learning or even misuse are prevented [12, 35].

Thus, we highlight suggestions aimed at improving navigation, such as the insertion of bars that show progress in the smartphone application and allow the user to map how much time will be spent to perform the task. In addition, the insertion of player buttons for the user to control audio, the inclusion of more back buttons in the app, and an increased size of the text boxes and font are relevant to the scores. As satisfaction with an application is associated with its use [36], we believe that an intuitive and user-friendly app is likely to be better used.

Other contributions pertained to colour semantics. There was feedback to improve visual features (e.g. removing red colours in the app and using lighter colours instead; using visual feedback for tasks already completed and to be completed). Additionally, other features of the interface were signalled to make it more pleasant. Some texts appeared “cut off” at their ends, and some buttons did not have centred icons.

Finally, informatics participants gave feedback to improve communication aspects. Suggestions were made regarding the app to provide the number of characters required in the password and pop-up windows to indicate the meaning of the items required to register and navigate.

Regarding usability, some suggestions were partially achieved. For feedback such as “At the assessment module, indicate how many questions there are and how many are left to the end,” we understood, based on another suggestion, that inserting a progress bar could solve the problem (i.e. time control spent on the activity).

In some feedback, we used other means to solve the underlying problem without fully implementing the informatics suggestion. For example, for the phrase “Put pop-up windows in the menu with topic information,” information that refers to the topic was described within this section without the creation of pop-up windows.

Regarding the feedback to improve the content of the “ + Adesão!” app, people living with HIV and health care workers made relevant comments. However, none of them individually served to adjust or insert any new behaviour change technique. The majority of health care workers’ suggestions focused on broader themes unrelated to adherence to medication (e.g. inserting content addressing the relationship between drug addiction and antiretroviral therapy use, inserting official links with general information about HIV and AIDS) or suggestions for features already covered by other apps (e.g. reminders to take medication).

Since the “ + Adesão!” app already provides texts and videos talking about HIV, we believe that it is not necessary at this time to provide external links and other materials. However, the authors do not exclude the possibility of implementing this in future versions of the app. Regarding the interaction of antiretroviral therapy and recreational drug use, content changes have not yet been made, mainly because this is a topic that requires care to avoid public stigmatization. Finally, we believe that the use of means to remember the timing of medicines is not relevant to the current work given that there are already other apps that perform this function [8].

Moreover, the interviewed patients suggested potential improvements to the app, such as inserting information about the daily life of those living with the virus (e.g. care to get a manicure) and the inclusion of links to specific social media and HIV + networks so that they can connect with others and vent emotions. All of these suggestions were of interest for future app versions. Currently, we aim to focus on adherence behaviours.

#### Usability elements

Overall, usability measures had high scores, which indicates that the app met the needs of potential users. It proved to be easy to use, with well-integrated functions that could dispense the external help of someone with technical knowledge without inconsistency and with little complexity. These elements contributed to the SUS score of 89, which demonstrates good usability [26]. Additionally, the app was satisfying, useful, easy to use and easy to learn.

Although people living with HIV made a few suggestions for improving the usability of the “ + Adesão!” app, the only critical usability assessments were “Placing the option ‘does not apply’ on the doctor–patient relationship issue list” and “Decreasing the amount of text and using more images”. The issue regarding the doctor–patient relationship was addressed, but other changes in the content (e.g. using more images) will be addressed in future developments.

One explanation for the few suggestions by patients on usability is that they perceived the app as easy to use and navigate. On the other hand, studies evaluating usability problems with end users and usability experts have shown that the latter group tends to report more interface-related problems [37]. Moreover, the suggestions made came from two participants who were HIV + and were university students, suggesting that education level may facilitate the elaboration of criticism.

#### Limitations

Some limitations must be noted regarding the methodology in terms of both content evaluation and usability. First, it should be noted that the data collection procedures differed between groups of experts, which may have affected the quantity and quality of the information obtained. The fact that informatics experts used the app alone and for a longer period of time, and therefore in more depth, may have contributed to the fact that they offered the highest number of suggestions, and these suggestions were more feasible in comparison to the suggestions of health care workers. We expected that health care workers would be able to provide more comments and pertinent suggestions regarding the content and techniques for behaviour change, mainly because they work routinely on HIV treatment adherence.

Regarding the usability measured with the participants living with HIV, it is essential to highlight that a research assistant was beside these participants during the use of the app. When the participants eventually requested clarification or tips on how to proceed with the use of the app, this assistance was provided. This situation may have facilitated the use of the “ + Adesão!” app such that criticisms regarding usability were suppressed. In addition, some of these participants sought to vent about their personal lives while using the app, which may have favoured social desirability when they responded to measures.

#### Conclusion

This study presented the development process of a new app tool following a person-based approach whose main feature is the participation of end users (people living with HIV), health care workers and information technology experts. All participants were able to share general perceptions and feedback to improve the app in terms of both usability and content. We consider the results to be satisfactory.

Despite some limitations, the “ + Adesão!” app is ready to be tested and evaluated in terms of benefits and results. As discussed, some suggestions were implemented and served as part of the basis for the development of version 2.0 of the app. Further studies need to address the feasibility and efficacy of the app to help people who have difficulties adhering to medical therapy for HIV.

## Study 2

### Feasibility and benefits study of an app intervention to help with adherence to antiretroviral therapy among people living with HIV

#### Methods

##### Design

A preexperimental study was conducted that comprised only one group that completed a pretest and posttest [25]. Mixed methods were used to evaluate the intervention’s outcomes. Before the study was developed, a sample calculation was performed according to Viechtbauer et al. [38]. The minimum sample size was estimated to be 14 participants. Participants were invited by convenience sampling, constituting a nonprobabilistic sample [25].

##### Participants

All participants came from an HIV and AIDS nongovernmental organization (NGO) located in the city capital area in southern Brazil. These were individuals in conditions of poverty and vulnerability who visited the NGO once a week. They were assisted by health services, such as medical consultations, psychological assistance and massage therapy. In severe cases, additional services were provided for personal well-being, such as hair cutting, food and bathing. Based on the NGO’s record of clinical data, history of treatment adherence and sociodemographic characteristics, we invited eighteen people living with HIV to participate in the study. Participants were recruited according to two main criteria: having a detectable viral load (*n* = 11) or having a history of treatment difficulties (e.g. missing doses, reporting no compliance, missing appointments, oscillating viral load) even if the viral load was undetectable at the beginning of the study (*n* = 4). In addition, three participants with an undetectable viral load and no confirmed history of treatment difficulties but in equal conditions of social exclusion and vulnerability were recruited since their feedback was equally important and of interest. Moreover, the participants were required to have a cell phone and know how to use it, be over 18 years of age, have been diagnosed with HIV for at least 6 months, and receive ART.

Ten participants were female and eight were male. The average age was 46.9 years (SD = 11.56), ranging from 31 to 70 years. On average, the participants had been living with HIV for 11.09 years (SD = 7.99) and had been using ART for 10.4 years (SD = 7.61). Regarding cell phone use, they reported using a cell phone 2.9 h a day on average (SD = 3.3) in addition to using an average of 3.67 apps (SD = 3.91).

##### Measures

Demographics

- *Sociodemographic questionnaire*—The sociodemographic questionnaire was developed ad hoc for this study. It included variables such as age, gender, education, income, race/ethnicity, psychiatric comorbidity, time with a cell phone, number of apps used and hours that the cell phone was used during the day. Additionally, health data were obtained from the clinical chart: time of HIV diagnosis and ART intake, CD4 lymphocyte count and viral load.

Primary outcome

- *Adherence to Antiretroviral Therapy: CEAT-VIH*—The CEAT-VIH [20, 21], validated for use in Brazil [39, 40], is a self-administered, fast and simple instrument consisting of 17 items (version 2) that assesses adherence to antiretroviral therapy from a multidimensional perspective [20, 21]. The instrument allows for calculating an overall measure of adherence called global adherence score (total score) and the following dimensions: compliance; antecedents of nonadherence behaviours; doctor–patient communications; personal beliefs and expectations about their treatment; and treatment satisfaction. The higher the score on the instrument, the greater the adherence to treatment. A recent international study with the version applied online showed good evidence of validity (e.g. validity related to an external clinical criterion, construct validity, factor invariance) and reliability for the instrument [39].

Secondary outcome

- *Well-being: WHO-5 (Five)*—The WHO-5 is a generic and short-term measure that assesses the level of emotional well-being over the past 14 days. Version 1, which was validated for use in Brazil [41], contains five items scored from 0 to 3 that provide a total score ranging from 0 to 15, with higher scores indicating greater well-being.

Intervention feasibility variables

- *Recruitability*—Recruitability indicates the ratio between participants eligible for the study who were invited and those who agreed to participate.
- *Attendance*—Attendance indicates the ratio between the predicted number of sessions of app use according to the number of participants and the number of sessions that the app was actually used.
- *Satisfaction: Satisfaction with the intervention*—This measure was adapted from Remor and Amorós-Gómez [42] to check the participants’ overall satisfaction with the intervention. It has nine questions with five ordinal response options, four dichotomous questions and a qualitative question in which the participant can include comments and observations.
- *Interview script for qualitative assessment*—The interview script was an ad hoc semistructured questionnaire developed for research purposes. It had questions to obtain feedback on aspects such as general perceptions of the intervention, the utility of the app, satisfaction, strengths, weaknesses and difficulties.

##### Data collection and procedures

After approval for the study was obtained from the Research Ethics Committee (CAAE: 14421219.5.0000.5334), an HIV and AIDS NGO was contacted to present the study and obtain consent to collect data onsite. The coordinator of the institution identified service users with a detectable viral load or with a history of problems adhering to HIV treatment (potential participants), presented a summary of the study and asked whether the researcher could personally carry out the invitation to participate in the research.

After each participant’s prior acceptance was given to the coordinator, the researcher presented the study in detail and obtained written informed consent. In total, 18 people living with HIV initially agreed to participate in the study and completed the sociodemographic data sheet, the CEAT-VIH and the WHO-5 at baseline (pretest). Paper-and-pencil versions of all instruments were used. Participants used the app on the exclusive day of the week that they attended the centre so that they did not have to come to the site only to participate in the research.

The intervention started on a different day of the evaluation and lasted for 4 weeks. Participants used the “ + Adesão!” app in a private and silent room with the presence of a trained researcher to answer questions and assist in using the app if necessary. Each session lasted an average of approximately 45 min. The participants performed two modules at a time in sessions 1, 2 and 3 and one module in session 4, totalling the seven modules of the app.

In the fourth week of the intervention, the posttest was performed with the application of the questionnaire’s outcomes. In addition, an external researcher without previous contact with the participants conducted a semistructured interview to measure satisfaction with the intervention and recorded the responses in audio format. Of the 18 participants who answered the pretest, 13 used the app at least once and completed the posttest.

##### Data analysis

Sociodemographic data, primary and secondary outcome quantitative measures, moderation of results and intervention viability were tabulated and analysed using the Statistical Package for Social Sciences software, version 18.0 (SPSS® Inc, Chicago, IL). Sociodemographic variables were summarized by means, standard deviations and frequencies according to specificity.

After assumption checks (test of normality, Shapiro–Wilks), to evaluate the preliminary results of the intervention on the adherence and well-being outcomes, a Wilcoxon nonparametric hypothesis test was performed using JASP version 0.16.3 (JASP computer software, Netherlands). It was used because it is appropriate for a few cases in repeated-measures studies [43]. In this case, we chose to include in the analysis only participants who participated in at least 51% of the intervention (*n* = 11). For the Wilcoxon signed rank test, the effect size (i.e. rank-biserial correlation) was calculated for all comparisons accompanying confidence intervals.

The feasibility measures of the intervention went through different statistical procedures. To assess recruitability, a “simple rule of three” was used that considered the number of people living with HIV who were invited to participate in the study and the number of those who accepted. Attendance also underwent a “simple rule of three” in which the number of existing sessions and the number of sessions that were held were considered. To determine satisfaction with the intervention, a frequency analysis was performed.

Finally, the qualitative data were transcribed and tabulated using NVivo software version 10 (QSR International Pty Ltd, Melbourne, Australia). We performed thematic analysis [44], which resulted in themes that described perceptions of the app.

#### Results

##### Benefits of using the app

According to the data in Table 6, after using the app for 4 weeks, differences were observed in the Global adherence score, i.e. the total score of the CEAT-VIH (effect size of 0.43), and perceived well-being (effect size of 0.45). However, the differences were not statistically significant. Observing scores individually, as shown in Table 7, six out of 11 participants improved their global adherence score, and one maintained the same score. Seven out of 11 participants improved their perceived well-being scores and one maintained the same score.

**Table 6:** Benefits of using the app: scores on adherence and well-being before and after app use (*N* = 11)

	Pretest	Posttest	Wilcoxon signed rank test		Effect size	95% CI for rank-biserial correlation	
Variable	Mean (SD), Mdn	Mean (SD), Mdn	*W*	*Z* (*p*)	Rank-biserial correlation	Lower	Upper
Compliance (CEAT-VIH)	13.18 (2.13), 13	13.09 (2.66), 14	27.000	− ﻿0.051 (1.00)	− 0.018	− 0.615	0.592
Antecedents of nonadherence (CEAT-VIH)	17.90 (2.16), 18	18.45 (2.01), 20	4.000	− 0.944 (0.410)	− 0.467	− 0.900	0.433
Treatment satisfaction (CEAT-VIH)	7.63 (2.24), 8	8.09 (1.86), 9	7.000	− 0.734 (0.520)	− 0.333	− 0.845	0.496
Doctor–patient communications (CEAT-VIH)	12.54 (3.07), 14	12.63 (2.46), 14	12.000	− 0.338 (0.792)	− 0.143	− 0.750	0.594
Personal beliefs and expectations about their treatment (CEAT-VIH)	21.00 (3.46), 20	22.18 (2.52), 23	11.000	− 1.362 (0.189)	− 0.511	− 0.862	0.169
Global adherence score (total score of the CEAT-VIH)	72.27 (8.84), 75	74.45 (8.21), 78	15.500	− 1.223 (0.240)	− 0.436	− 0.823	0.227
Perceived well-being (WHO-5)	8.63 (4.52), 8	10.36 (4.31), 12	15.000	− 1.274 (0.220)	− 0.455	− 0.830	0.206

*Note*: The rank-biserial correlation (*r*_*B*_) can be considered as an effect size and is interpreted the same as Pearson’s *r*. The *r* value varies from 0 to close to 1. The interpretation values for *r* commonly in published literature and on the internet are as follows: 0.1 – < 0.3 (small effect), 0.3 – < 0.5 (moderate effect) and ≥ 0.5 (large effect). *Mdn* = median, *CI*  confidence interval

**Table 7:** Qualitative representation of individual benefits of using the app: scores before and after app use

		Participants											
Variables		1	2	3	4	5	6	7	8	9	10	11	Number (%) of participants that improved
Compliance (CEAT-VIH)	Pretest	15	13	15	8	14	13	13	15	13	11	15	4 out of 11 (36%)
	Posttest	14	15	14	7	11	15	15	15	15	10	13	
Antecedents of nonadherence (CEAT-VIH)	Pretest	17	19	18	14	20	15	18	16	20	20	20	3 out of 11 (27%)
	Posttest	16	19	20	15	20	20	17	16	20	20	20	
Treatment satisfaction (CEAT-VIH)	Pretest	9	2	8	8	7	8	7	6	10	9	10	4 out of 11 (36%)
	Posttest	9	6	9	4	7	9	7	10	9	9	10	
Doctor–patient communications (CEAT-VIH)	Pretest	15	6	14	9	11	14	14	15	15	10	15	2 out of 11 (18%)
	Posttest	14	8	14	11	11	14	15	15	14	9	14	
Personal beliefs and expectations about treatment (CEAT-VIH)	Pretest	25	16	20	17	21	20	19	25	19	25	22	7 out of 11 (64%)
	Posttest	25	20	21	18	19	21	24	25	23	23	25	
Global adherence score (total score of the CEAT-VIH)	Pretest	81	56	75	56	73	70	71	77	77	75	82	6 out of 11 (54%)
	Posttest	78	68	78	55	68	79	78	81	81	71	82	
Well-being (WHO-5)	Pretest	14	3	14	12	9	6	4	3	15	8	7	7 out of 11 (64%)
	Posttest	15	1	14	5	10	12	12	7	14	12	12	

##### Intervention feasibility

The ratio between invited participants who met the eligibility criteria and those who ultimately accepted it was 95%. Twenty patients were invited, and 18 accepted Table 8 indicates attendance at the intervention session to use the mobile application. Of the 18 participants able to use the “ + Adesão!” app, 15 used the app at least once. Seven attended all sessions (100%), four attended 75% of sessions, three attended 25% of sessions and one participant attended 50% of sessions. Considering the subgroups of participants, the attendance of those with a detectable viral load was 32.27%, compared with 91.67% of those with only an undetectable viral load and 100% of those with an undetectable viral load and a history of difficulty in treatment. In total, 72 sessions of using the app were foreseen; there were 45 sessions with a total attendance of 62.5%.

**Table 8:** Attendance at the intervention session to use the mobile application

Participant	Session 1	Session 2	Session 3	Session 4	Attendance (%)
1	X	X	X	X	100
2	X	X	X	X	100
3	X	X	X	X	100
4	0	0	0	0	0
5	0	X	X	X	75
6	X	0	0	0	25
7	0	0	0	X	25
8	X	X	X	X	100
9	X	X	X	X	100
10	X	X	X	X	100
11	X	X	0	X	75
12	0	0	X	0	25
13	0	X	X	X	75
14	0	0	X	X	50
15	0	0	0	0	0
16	X	X	X	X	100
17	0	X	X	X	75
18	0	0	0	0	0
Total					62.5%

*Note*: X = attendance at the session, 0 = absence at the session

Table 9 describes the features of satisfaction with the intervention. In general, most reviews regarding the “ + Adesão!” app were positive. All participants felt well or very well during the intervention, and all stated that they were at least satisfied or very satisfied with the app and the information it provided. Only one participant (7.7%) found it difficult to complete the “ + Adesão!” modules, but all participants reported understanding the content covered. Regarding the length of the modules, most found them to be neither long nor short (*n* = 7; 53.8%).

**Table 9:** Satisfaction with intervention: descriptive

Item	*N* = 13	%
In general, how did you feel during the intervention?		
Well	7	53.8
Very well	6	46.2
What is your overall satisfaction with the intervention?		
Satisfied	5	38.5
Very satisfied	8	61.5
What is your satisfaction with the learning provided by the intervention?		
Satisfied	6	46.2
Very satisfied	7	53.8
In your case, was it difficult to complete all the intervention modules?		
No	12	92.3
Yes	1	7.7
In general, did you understand the contents and themes covered in the modules?		
Yes	13	100
How did you perceive the modules?		
Long	4	30.8
Neither long nor short	7	53.8
Short	1	7.7
Very short	1	7.7

##### Qualitative perceptions of “ + Adesão!”

After the application of the thematic analysis, four main themes were generated in relation to perceptions of the “ + Adesão!” app: (a) the app as a motivational tool in treatment; (b) an informational app; (c) a means of venting; and (d) aspects to be improved. These themes indicated the acceptability and importance of the tool in addition to noting aspects that could be improved in the future.!”

- *The app as a motivational tool in treatment*: Some participants reported that the app could improve aspects related to motivation in treatment, which was one of the main benefits of its use. This feature would be taken into account when participants recommended the use of the app to someone else.

“I would recommend the app because it cheers you up. It gives you a boost, something different.” (Man, 58 years old).

“It (the app) brings some questions that give a, well… give a greater meaning so that we can do the treatment with a certain seriousness.” (Man, 32 years old)

(b) *Informational app*: Many participants saw the information they received as a great virtue of the app in relation to HIV, the treatment and the medications. Even those who already knew a large amount of information about the virus and the treatment mentioned that they had gained new knowledge.

“For me, it was great. I learned a lot of things about my treatment. A lot of things that I didn’t know I now know.” (Woman, 47 years old).

“The app is really clarifying, taking away doubts about how people live with the medicine, how they maintain the treatment, take it, adapt, get used to it. It’s a partner for life, right?” (Woman, 58 years old).

“I found it interesting that we learn. I learned many things that I didn’t know about the disease.” (Woman, 39 years old).

(iii) *Means of venting*: The app was seen as a tool that can help individuals vent emotions and thoughts about HIV. It was indicated as a useful tool for people who may have more difficulty interacting with peers and talking about their health. In addition, the role that venting emotions can play in accepting the diagnosis was indicated.

“It is a work that encourages people to talk about their coexistence with HIV, which many people, even though they live with it, say they accept it and don't accept it. Many times the family doesn't accept it, so it is a way for people to bring out that… that feeling they have of rejection, of family disregard, of abandonment. And this makes people see that it is a normal thing, like diabetes, like a heart problem, you know?” (Woman, 58 years old).

“It’s like a … like a book, you know? Like that, you don't have anything to do, those books (journals) we used to do when we were little. When you were fighting with your mother or with your family and couldn't let go of that anger, you wrote all that stuff down. An outburst." (Woman, 38 years old).

(iv) *Aspects to be improved*: Finally, this category refers to the response patterns that noted constructive criticisms regarding the app. Participants referred to the content and, to a lesser extent, some aspects of usability. A video in the English language, despite having subtitles, was noted as an obstacle. In addition, improvements to the app’s aesthetics were suggested.

“I thought it should be in Portuguese (the video). Why? I hadn't seen that video yet. I knew how HIV spreads inside people, but not in the video. So, the video was much more educational.” (Woman, 49 years old).

“The app should be more eye-catching to call attention. It's like carnival; when we go to carnival, we make the costumes ourselves. It's all kinds of colours, it's beautiful, it's drawing, the glitter, one by one, sewn with love and care. So, it is different. Now, if you put only one or two colours, it makes no sense, there is no design or anything to call attention, then it is no fun. It's just a direct and straightforward letter.” (Woman, 36 years old).

“The weakness, for me, was that the video was in English and not in Portuguese.” (Woman, 38 years old).

#### Discussion

Before conducting studies with robust designs that consume more time and resources, such as randomized clinical trials, pilot studies have been suggested [12, 23] as a means of prior preparation. Thus, in addition to checking the benefits of an intervention or programme, we aimed to evaluate variables that may impact the intervention’s effectiveness in the future, such as attendance, recruitment, satisfaction and acceptability. Therefore, the present study sought to verify the conditions to obtain more solid evidence about the capacity of the “ + Adesão!” app to assist in improving adherence considering the perspective of its end users.

At the outset, it should be noted that, if the use of the app did not present any immediate benefit in improving adherence to ART, it did not cause damage (e.g. decreasing well-being). A possible explanation for this result includes the time spent using the app, the format of the intervention and some methodological limitations, such as the application of the posttest on the same day that the last module was performed.

The smartphone application used for 4 weeks (dose) may have been insufficient to cause changes in the reported adherence to ART. App-based interventions that have sought to change behavioural aspects in addition to cognitive and emotional components have varied in length, lasting for at least 3 months and up to 1 year (e.g. [16]). As the aim is to acquire new skills in addition to changing dysfunctional patterns in behaviour, the intervention time must be longer. In addition, it is estimated that attendance in the intervention may be related to the benefits of adhering to treatment as the participants attended only 62.5% of the planned sessions. Perhaps the benefits would be more evident given greater attendance.

Another source of difficulty was related to the use of the app. The “ + Adesão!” app had seven modules, and a 4-week intervention format was chosen. Thus, participants completed two modules in the first 3 weeks and only one module in the last week. Although the assessment of satisfaction with the intervention did not indicate problems in the extension and understanding of the modules, it can be hypothesized that the accomplishment of two modules per session had an impact on learning due to the amount of information that was addressed.

Finally, related to the application of the posttest protocol, it is worth mentioning that since the centre where the data collection was conducted was about to end its activities for the season, there was no time to administer the posttest the following week, as expected. Thus, some changes in adherence and well-being may have occurred, but they were not identified.

On the other hand, analysing the use of the application at the individual level, the number of participants who improved their adherence to antiretroviral therapy and well-being was higher than the number of those whose adherence worsened (6 versus 4 and 7 versus 3, respectively). This may suggest that using the app may be beneficial according to the user’s profile. However, further data are necessary.

#### Intervention feasibility variables

The recruitability of the intervention was perceived as high; only two people living with HIV out of the 20 invited to participate declined participation, constituting 95% recruitability. These data are superior to those of a recent study in which 67.7% of the invited participants agreed to use an application to improve adherence to ART over 3 months [45]. Since the present study took 4 weeks, it is estimated that time may be a variable that influenced recruitment.

Despite the high recruitability, attendance in the app’s use proved to be below expectations, with a rate of only 62.5%. Other feasibility studies in the context of adherence to ART have shown attendance higher than 90% (e.g. [46]). However, it must be considered that in the country where the present research was conducted, payment for participation in studies is forbidden, which tends to undermine attendance. Moreover, the study participants were recruited from a vulnerable population. Poor attendance may be attributed to structural barriers such as lack of income, which could potentially be linked to a design flaw of the study: participants without the means to access the NGO were required to travel to the NGO to complete app sessions.

Regarding people with a detectable viral load, the main targets of the study, attendance was even lower (32.5%). There is evidence that hard-to-reach individuals may have the worst control of the disease (e.g. detectable viral load results), since competing subsistence needs may supersede health care retention [47], suggesting the need for differentiated strategies for this audience. In this sense, conducting the intervention in specific locations or even in the laboratory may not be the most suitable approach for people living with HIV with a detectable viral load, which suggests the need for future tests of the “ + Adesão!” app in natural contexts. One of the chief virtues of mHealth in the context of HIV is to serve as an option for people with difficulties attending face-to-face interventions [46].

Finally, the intervention was viewed positively by the participants, who reported prominent scores in terms of satisfaction with the intervention in addition to providing feedback about the application that indicated its acceptability and utility. Since satisfaction with the app is linked to its future use, the assessment of the app looks promising. These data complement previous evidence about the “ + Adesão!” app that indicated good indices of usability measures.

As only one participant found it difficult to finalize the intervention modules and all participants understood the content (100%), there was no need to make immediate changes to the content. In addition, although 4 participants considered the modules long (30.8%), not limiting the time to complete the intervention may help with this perception. The changes that will be made in the app will be mainly due to qualitative feedback, such as the insertion of more colours and the substitution of a video subtitled in Portuguese to a video in the Portuguese language.

#### Limitations

Some limitations should be noted, especially concerning the sample and the setting of the app’s test. Although the site where the data were collected has many users with a detectable viral load, access to these participants is complicated since they usually attend the centre less frequently. Another limitation is that the participants used the app on the day they regularly visited the centre. It is impossible to estimate whether the attendance would be the same if the participants’ only reason to come was exclusively to use the app.

Furthermore, it is noteworthy that a researcher accompanied the participants during the use of the “ + Adesão!” app. The participants tried to interact and talk about personal life problems while using the app, and on some occasions, the researcher had to explain the meaning of some words or indicate how to proceed with the app’s use. These interactions may have fostered a positive perception of the experience.

#### Conclusion

Study 2 described the process of evaluating the feasibility and benefits of using a smartphone application to improve adherence to ART. The results regarding the app were promising, showing the potential for its effectiveness and allowing further controlled studies with the tool since the study was able to assess the potential for recruitability, attendance, satisfaction and general positive perceptions regarding the use of the “ + Adesão!” app. Moreover, the use of the app did not reduce perceived well-being. However, according to late feedback, it is important to make adjustments to the app to make it feasible for users to use the app independently.

## General discussion

This article covered studies that presented the creation process, theoretical guided development and preliminary evaluation of the “ + Adesão!” smartphone application in the context of health care with participants in conditions of poverty and vulnerability. Furthermore, we followed relevant methodological procedures, such as the study of the usability and content of the app and necessary adjustments concerning these elements (Study 1). Obtaining evidence of feasibility for a future intervention is linked to the recruitment variables, attendance, satisfaction with the intervention and other perceptions of the app (Study 2).

It is important to highlight that the application proved to be easy to use by end users (people living with HIV) and was perceived as helpful in assisting them with their treatment adherence. The ease of use contradicted the prediction of the experts consulted who indicated the possibility of the app being challenging to use. However, it should be noted that in both studies, participants living with HIV were accompanied by a research assistant who addressed the participants’ doubts and helped whenever necessary. We highlight the importance of future studies in natural contexts to evaluate the use of the app without skilled support.

Regarding the app’s content, the feedback received contributed to its improvement. Although the health care workers and informatics experts consulted experienced difficulty evaluating active components of the app, which may be related to a lack of knowledge about cognitive-behavioural approaches, it led the authors to be cautious with this specific feedback.

On the other hand, we found no statistically significant differences in outcomes (i.e. adherence to treatment and emotional well-being). These results may be related to the short time spent using the app and the failure to complete the posttest 1 week after the end of the intervention. However, the qualitative feedback showed mostly positive comments that attested to its applicability, and most participants presented prominent scores in terms of satisfaction with the intervention. In addition, the intervention proved to be viable since it had high recruitability, although attendance was below expectations, especially among participants with a detectable viral load.

Regarding feasibility measures, recruitment for the intervention proved to be high since only two people declined the invitation to participate, which shows a genuine interest in using the app. Despite the high recruitability, however, attendance at the intervention sessions was only 62.5%. In the case of participants with a detectable viral load, attendance was lower; they attended only 32.5% of the planned sessions. However, it is worth mentioning that the participants were persons in conditions of poverty and high vulnerability, which increases barriers to treatment. These results suggest the importance of further trials with the app, including the selection and participation of patients with the worst control of the disease.

According to the questionnaires, participants reported high levels of satisfaction with the intervention and seemed to be satisfied with it. In the same way, numerous positive comments were made about the app that indicated that it could be a motivational tool in treatment and a means of obtaining information about HIV and AIDS and encouraging users to vent emotions about the virus and disease.

The “ + Adesão!” app is now in an advanced development process. Beyond the qualitative notes made in study 2, subsequent steps will include introducing further improvements to the app to make it ready for inclusion in further studies to assess its benefits and efficacy in more controlled trials.

## Supplementary Information

**Additional file 1.** TheTIDieR (Template for Intervention Description and Replication) Checklist.

## Data Availability

The datasets generated and/or analysed during the current study are not publicly available due to the personal and/or qualitative nature of the information provided by the research participants but are available from the corresponding author on reasonable request.
