# Supplementary material for: Development, feasibility testing and perceived benefits of a new app to help with adherence to antiretroviral therapy in people living with HIV in Brazil
Source: Pilot Feasibility Stud. 2023 Jul 26;9:130. doi: 10.1186/s40814-023-01370-7 (PMC10369752; doi:10.1186/s40814-023-01370-7)
Supplement: Supplementary file 1 — Additional file 1. TheTIDieR (Template for Intervention Description and Replication) Checklist. [file 40814_2023_1370_MOESM1_ESM.docx]

**
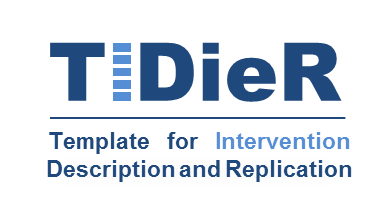
The TIDieR (Template for Intervention Description and Replication) Checklist*:**

Information to include when describing an intervention and the location of the information

| **Item number** | **Item** | **Where located **** | |
| --- | --- | --- | --- |
|  |  | Primary paper  (page or appendix  number) | Other ^†^ (details) |
|  | **BRIEF NAME** |  |  |
| **1.** | "+Adesão!", in the original Portuguese; meaning plus adherence. It is a new smartphone application developed in the Brazilian cultural context to help improve compliance related to antiretroviral treatment. | Pages 5 and 6 | ______________ |
|  | **WHY** |  |  |
| **2.** | We propose a digital intervention aiming to improve adherence to ART based on a cognitive-behavioral approach that focuses on activities in the domains of compliance, antecedents of nonadherence behaviors, doctor-patient communication, personal beliefs/expectancies about the treatment, and treatment satisfaction. These domains have been reported as related to adherence behavior. | See Table 1 | Pages 5 and 6 |
|  | **WHAT** |  |  |
| **3.** | The intervention is delivered by a mobile application to improve adherence to ART based on a cognitive-behavioral approach. It comprises seven modules tailored according to a pre-evaluation of the adherence levels (see Table 1 for module titles, objectives, and active components based on BCT Taxonomy). In addition, the app prioritizes areas that are more relevant to the patient. | Pages 5 and 6 | See Table 1 |
| **4.** | The contents for each domain/session are different; they focus on specific activities to develop a particular skill related to adherence to treatment. E.g., Start becoming aware of adherence to therapy through self-evaluation by a validated measure. App gives feedback on behavior and proposes an itinerary of modules based on the aspects that are relevant to the user. Including, for example, information about HIV infection, information on the importance of treatment adherence, and reflective exercises that address treatment beliefs and expectations related to medicines. In addition, it presents activities that address communication with healthcare professionals. And an exercise on how to deal with unforeseen events in the treatment. Finally, it concludes with feedback on the level of adherence to therapy after the modules are completed. | Pages 5 and 6 | See Table 1 |
|  | **WHO PROVIDED** |  |  |
| **5.** | Mobile application. All activities are programmed with the intention of not requiring the presence of a healthcare professional. | Pages 5 and 6 | _____________ |
|  | **HOW** |  |  |
| **6.** | The mobile application does not require an internet connection all the time. The user has the option to send exercises and activities to email. To be used individually. | Pages 5 and 6 | _____________ |
|  | **WHERE** |  |  |
| **7.** | Mobile application. Smartphone. | Pages 5 and 6 | _____________ |
|  | **WHEN and HOW MUCH** |  |  |
| **8.** | The app intervention includes seven modules (sessions) that the user schedules its development and completion according to their time. Modules are developed with activities that employ at least 30 minutes. Therefore, it is recommended to complete one to two modules by week. | Pages 5 and 6 | _____________ |
|  | **TAILORING** |  |  |
| **9.** | The App intervention was planned to be personalised. It comprises seven modules tailored according to a pre-evaluation of the adherence levels. | Pages 5 and 6 | _____________ |
|  | **MODIFICATIONS** |  |  |
| **10.^ǂ^** | The intervention was modified during the process of development and study through feedback from patients, healthcare providers, and informatics experts. The research team integrates all information provided to improve the app content, aesthetics, and usability. All details on modification are reported in studies 1 and 2. | All manuscript | _____________ |
|  | **HOW WELL** |  |  |
| **11.** | Planned: Completion of two modules by week was planned, and 100% of assiduity was expected. | Study 2 | Table 8 |
| **12.^ǂ^** | Actual: Completion of two modules by week was delivered as planned. However, 62,5% of assiduity was observed. | Study 2 | Table 8 |

† If the information is not provided in the primary paper, give details of where this information is available. This may include locations such as a published protocol or other published papers (provide citation details) or a website (provide the URL).

ǂ If completing the TIDieR checklist for a protocol, these items are not relevant to the protocol and cannot be described until the study is complete.

* We strongly recommend using this checklist in conjunction with the TIDieR guide (see *BMJ* 2014;348:g1687) which contains an explanation and elaboration for each item.

* The focus of TIDieR is on reporting details of the intervention elements (and where relevant, comparison elements) of a study. Other elements and methodological features of studies are covered by other reporting statements and checklists and have not been duplicated as part of the TIDieR checklist. When a **randomised trial** is being reported, the TIDieR checklist should be used in conjunction with the CONSORT statement (see [www.consort-statement.org](http://www.consort-statement.org)) as an extension of **Item 5 of the CONSORT 2010 Statement.** When a **clinical trial** **protocol** is being reported, the TIDieR checklist should be used in conjunction with the SPIRIT statement as an extension of **Item 11 of the SPIRIT 2013 Statement** (see [www.spirit-statement.org](http://www.spirit-statement.org)). For alternate study designs, TIDieR can be used in conjunction with the appropriate checklist for that study design (see [www.equator-network.org](http://www.equator-network.org)).
